# Impact of cultivars, nitrogen rates, and sugar beet processing by-product on wheat growth and yield

**DOI:** 10.1186/s12870-025-07560-0

**Published:** 2025-11-14

**Authors:** Asaad Reda Ibrahim, Mohamed El Sayed Moursy, Ahmed M. Abdelghany, Sobhi F. Lamlom

**Affiliations:** 1 Wheat Research Department, Agricultural Research Center, Field Crops Research Institute, Giza, 12619 Egypt; 2 Soil Fertility and Plant Nutrition Department, Agricultural Research Center, Soil, Water and Environment Institute, Giza, 12619 Egypt; 3 Crop Science Department, Faculty of Agriculture, Damanhour University, Damanhour, 22516 Egypt; 4 Plant Production Department, Faculty of Agriculture Saba Basha, Alexandria University, Alexandria, 21531 Egypt

**Keywords:** Integrated nutrient management, Sustainable agriculture, Sugar beet filter cake (BFC), Organic amendments

## Abstract

The wheat production management in the newly reclaimed soils requires innovative approaches that combine organic amendments with traditional fertilization methods. This research examined the combined effects of sugar beet filter cake (BFC) (without treating and 24 t ha⁻¹) and mineral nitrogen fertilization rates (60, 120, 180, and 240 kg N ha⁻¹) on the agronomic performance and yield components of three bread wheat cultivars (Sakha 95, Misr 3, and Sids 14) in the newly reclaimed calcareous soils across two growing seasons (2021/2022 and 2022/2023). A split-plot design with three replications assessed ten essential agronomic traits. Analysis of variance indicated significant seasonal effects (*p* ≤ 0.05) on physiological traits, with SPAD values declining from 40.92 to 25.61 across seasons. Fertilization treatments were the most significant factor, demonstrating highly significant effects (*p* ≤ 0.001) on 90% of the assessed parameters. Cultivar Sakha 95 exhibited enhanced grain yield (7.47 t ha¹) and harvest index (38.34%), whereas Sids 14 showed optimal morphological characteristics, including maximum plant height (114.15 cm) and flag leaf area (43.43 cm²). Combining BFC with 240 kg N ha⁻¹ resulted in the highest grain yield (8.48 t ha⁻¹) and spikes per square meter (340.61), indicating increases of 52.5% and 28.9% compared to the lowest nitrogen rate, respectively. Path coefficient analysis revealed that biological yield and harvest index are the main determinants of yield, with coefficients of 0.813*** and 0.614***, respectively. Principal component analysis accounted for 93.81% of the total variation (PC1 = 80.15%, PC2 = 13.66%), indicating clear clustering patterns between organic and non-organic fertilization regimes. The findings offer insights into optimizing integrated nutrient management strategies to enhance wheat productivity in newly reclaimed soils.

## Introduction

 Wheat (*Triticum aestivum* L.) is one of the most widely grown crops in the world and holds a crucial social and economic position [1]. Due to wheat’s significant nutritional requirements as a cereal crop, appropriate fertilization is crucial for attaining optimal yields. Nitrogen (N) is the most critical macronutrient determining wheat productivity and grain quality. It is a cornerstone for several key physiological processes directly impacting grain yield formation. Primarily, nitrogen is essential for photosynthetic efficiency through chlorophyll synthesis, with research demonstrating that adequate N supply can increase leaf chlorophyll content by 30–40%, extending the photosynthetically active period of flag leaves during grain filling [2, 3]. Additionally, nitrogen significantly influences tillering capacity and spike formation, which are primary yield components [4]. Studies have shown that optimizing N application can increase productive tiller number by 15–25% compared to deficient conditions, directly translating to higher grain yield potential [5]. Nitrogen also enhances spikelet differentiation and reduces floret abortion, potentially increasing kernels per spike by up to 30% under optimal conditions versus N-limited environments [6]. Research has consistently demonstrated that nitrogen deficiency can reduce wheat grain yields by 20–50%, depending on the severity and timing of limitation [7]establishing it as the most yield-limiting factor in non-fertilized wheat production systems. Consequently, strategic nitrogen management remains central to achieving economically viable wheat yields while maintaining grain quality standards demanded by processors and consumers.

Despite enhancing crop performance, N fertilizers can negatively impact soil health when used improperly [8]. The long-term and excessive application of mineral N fertilizers to increase yields has raised significant concerns about agricultural sustainability [9, 10]. While these fertilizers have substantially boosted crop production, research indicates that approximately 45% of applied N is not absorbed by crops and is subsequently lost from the soil [11, 12]. This inefficiency not only increases production costs but also contributes to soil degradation, water pollution, greenhouse gas emissions, and soil acidification, raising serious questions about the environmental consequences of current fertilization practices [13, 14]. Mismanagement of fertilizers harms the environment and public health. Therefore, effective N management is crucial for improving N use efficiency, increasing crop yields, and mitigating the negative impacts of N misuse, which remains a global concern [15].

In addressing these challenges, agricultural researchers and farmers are progressively adopting organic amendments as a sustainable alternative that aligns with environmental considerations. Organic fertilizers, which are abundant in nutrients and organic matter, have the potential to enhance soil fertility and improve the efficiency of fertilizer use, while also reducing the adverse environmental effects linked to traditional nitrogen fertilization methods [16, 17]. As such, organic amendments present a promising alternative to chemical fertilizers [18, 19], though they may not be sufficient on their own to sustain high crop yields due to their lower nutrient content and slower release rates [10, 20]. To address this, some studies have explored the partial substitution of chemical fertilizers with organic alternatives [21, 22]. Among the various options for organic fertilizers, agricultural waste products have received significant attention. Sugar beet filter cake (BFC), generated as a byproduct of the sugar industry, serve as a particularly advantageous source of organic fertilizer [23]. The BFC are prevalent in numerous agricultural areas and contain significant amounts of essential nutrients and organic matter, positioning them as suitable options for sustainable fertilization methods [24, 25].

Sugar beet filter cake (BFC) represents a valuable agricultural resource with significant potential for enhancing soil fertility in degraded or newly reclaimed soils. However, nutrient availability from organic amendments follows distinct mineralization patterns that differ from the immediate availability found in mineral fertilizers. Based on incubation studies with similar materials, Roig et al. [26] reported that N mineralization from sugar processing by-products follows a biphasic pattern, with approximately 20–25% of total nitrogen becoming plant-available within the first 30 days after incorporation, followed by a slower release of an additional 15–20% over the subsequent 60–90 days. In addition to the nutrient contributions from the first year mentioned above, BFC also serves as a valuable source of phosphorus and organic matter, despite its high moisture content. Applying BFC to soil has been identified as a practical approach [27]. Sugar beet filter cake can help reduce nutrient pollution through several mechanisms: (1) improving soil structure and water-holding capacity, which decreases nitrate leaching; (2) providing organic forms of nutrients that are gradually released through microbial mineralization, better aligning with plant uptake patterns; and (3) enhancing soil microbial biomass, which can temporarily immobilize excess nutrients during periods of low plant demand. These processes collectively contribute to greater nutrient retention in the root zone and reduce transport to groundwater and surface water [28–31]. Also, this byproduct has been widely used as a complete or partial replacement for mineral fertilizers in various crops, including sugar beet [27, 32–34]. The production quantity of sugar beet in Egypt rose from 7.84 to 14.83 million metric tons between 2010 and 2021 [35], increasing the residual and waste quantities generated at each stage of sugar manufacturing from sugar beet. The leftover sugar beet is the naturally released suspended solids that are made during the washing process. These solids are separated into a water layer and a tare layer, and then they are collected in basins to dry in the sun. This leftover material is mostly the fertile soil that sticks to the sugar beet root and the rootlets that grow on top of it. It is high in organic matter and has a lot of macro and micro plant nutrients [36].

This study addresses the underexplored potential of BFC combined with different wheat cultivars and nitrogen fertilization rates. Responding to challenges of soil fertility maintenance and rising mineral fertilizer costs, it examines how BFC and mineral nitrogen affect wheat production. We hypothesized that (1) the integration of BFC with mineral nitrogen would result in synergistic effects on wheat productivity compared to sole mineral nitrogen application; (2) the magnitude of this synergistic effect would vary with nitrogen application rate, with greater benefits at intermediate rates; and (3) wheat cultivars would respond differently to these integrated nutrient management strategies based on their inherent nitrogen use efficiency traits.”

## Materials and methods

### Experimental conditions

A field experiment was carried out on the research farm of the Nubaria Agricultural Research Station, ARC (30° 52’ 56’’ N Latitude and 29° 58’ 01’’ E Longitude) over two consecutive seasons (2021/2022 and 2022/2023).The region experiences a Mediterranean-type climate characterized by mild winters and hot summers (Table 1). Winter months (December-February) showed average temperatures ranging from 12 to 15 °C, while spring months (March-May) displayed a gradual warming trend with temperatures rising from approximately 16 °C to 23 °C. Maximum temperatures rarely exceeded 30 °C during the winter growing period, providing favorable conditions for the cultivation of winter crops. Rainfall in the Nubaria region follows the typical Mediterranean distribution pattern with most precipitation occurring during winter months (November-February).

**Table 1:** Monthly meteorological parameters recorded at Nubaria agricultural research station during two growing seasons (2021–2023)

Month	Temperature (°C) − 2021/2022			Temperature (°C) − 2022/2023			Precipitation (mm)		Relative Humidity (%)	
	Max	Min	Avg	Max	Min	Avg	2021/2022	2022/2023	2021/2022	2022/2023
December	~ 25	~ 8	~ 15	~ 24	~ 8	~ 14	~ 12	~ 15	~ 70	~ 73
January	~ 22	~ 6	~ 12	~ 21	~ 5	~ 11	~ 18	~ 20	~ 75	~ 78
February	~ 23	~ 7	~ 13	~ 22	~ 6	~ 12	~ 15	~ 17	~ 72	~ 75
March	~ 25	~ 9	~ 16	~ 24	~ 8	~ 15	~ 10	~ 12	~ 65	~ 68
April	~ 28	~ 12	~ 20	~ 27	~ 11	~ 19	~ 5	~ 6	~ 60	~ 63
May	~ 30	~ 15	~ 23	~ 29	~ 14	~ 22	~ 2	~ 3	~ 55	~ 58
Seasonal Total/Average	~ 25.5	~ 9.5	~ 16.5	~ 24.5	~ 8.7	~ 15.5	~ 62	~ 73	~ 66.2	~ 69.2

Physicochemical analysis of the soil at the experimental site during the study seasons are shown in Table 2. Soil samples were taken from each experimental plot at two depths (0–30 cm and 30–60 cm) using a spiral auger of 2.5 cm diameter. Five subsamples were collected in a systematic zigzag pattern across each plot and combined to form a composite sample for each depth and plot. Baseline sampling was conducted before treatment application in November 2021 (pre-planting of the first season) to establish initial soil conditions, with follow-up sampling conducted in October 2022 (prior to the second season) to assess changes in soil properties. The samples were air-dried at 40 °C, ground to pass through a 2-mm sieve, and stored in labeled plastic bags until analysis. The organic matter content and soil N content were determined through wet oxidation determination and the Kjeldhal method, respectively [37]. The phosphorus and potassium contents were determined by spectrophotometry and flame photometer, respectively. Soil texture was determined using the hydrometer method, while pH and EC were measured in soil-water suspensions (1:2.5 and 1:5, respectively). The analyses were conducted at Soil, Water and Environment Research Institute (SWEI), Agricultural Research Center, 12,619, Giza, Egypt. The treatment-specific sampling approach allows us to detect potential differences in soil properties resulting from the different fertilization treatments over the course of the study.

**Table 2:** Physical and chemical properties of the soil at the experimental site

Chemical properties		Season	
		2021/2022	2022/2023
Ec (ds/m)		2.11	2.39
PH		8.13	8.35
Soluble cations meq/L	Ca^++^	7.26	7.66
	Mg^++^	2.07	2.58
	Na^+^	8.97	10.8
	K^+^	2.8	2.86
Soluble anions meq/L	CO3^−−^	-	-
	HCO3^−^	3.58	5.19
	Cl^−^	10.95	11.33
	SO4^−−^	6.57	7.38
CaCo3%		22.92	23.82
Organic M. %		0.24	0.31
Partical size distribution	Sand %	58.39	62.93
	Silt %	16.51	11.04
	Clay %	25.10	26.03
Soil Texture		Sandy Clay Loam	Sandy Clay Loam
Available macronutrients (ppm)	N	61.77	64.25
	P	3.51	3.82
	K	180.1	186.2
Available micronutrients (ppm)	Fe	1.55	2.00
	Cu	1.07	1.11
	Zn	0.43	0.33
	Mn	1.65	1.33

### Treatments and experimental design

The experiment was conducted using a split-plot design with three replications. Main plots (cultivars) were randomized within each of the three complete blocks, and sub-plot treatments (nitrogen × filter cake combinations) were randomized within each main plot. Blocks were arranged perpendicular to the prevailing slope of the experimental area to account for potential soil fertility gradients. The plot area of 5.6 m2 consisted of 8 rows, 3.5 m long and 20 cm apart. Three wheat cultivars (Sakha 95, Misr 3, and Sids 14) were assigned to the main plots. The three wheat cultivars selected for this study represent various genetic backgrounds and adaptation traits relevant to Egyptian agriculture. These cultivars were chosen based on their differing nitrogen use efficiency characteristics observed in preliminary screening, varying growth habits, and widespread adoption in newly reclaimed lands. Including multiple cultivars allows for the assessment of genotype-specific responses to integrated nutrient management, essential for developing targeted fertilization recommendations for farmers in the region. The sub-plots consisted of factorial combinations of nitrogen fertilization rates and BFC applications. Four nitrogen rates (60, 120, 180, and 240 kg N ha⁻¹) were applied in combination with one application level of BFC(24 t ha⁻¹) and a control treatment without organic amendment (0 t ha⁻¹), resulting in the following eight treatment combinations:

1. 60 kg N ha⁻¹ + 24 t ha⁻¹ BFC (T1).
2. 120 kg N ha⁻¹ + 24 t ha⁻¹ BFC (T2).
3. 180 kg N ha⁻¹ + 24 t ha⁻¹ BFC (T3).
4. 240 kg N ha⁻¹ + 24 t ha⁻¹ BFC (T4).
5. 60 kg N ha⁻¹ + 0 t ha⁻¹ BFC (T5).
6. 120 kg N ha⁻¹ + 0 t ha⁻¹ BFC (T6).
7. 180 kg N ha⁻¹ + 0 t ha⁻¹ BFC (T7).
8. 240 kg N ha⁻¹ + 0 t ha⁻¹ BFC (T8).

The physicochemical properties of the BFC used are presented in Table 3. Each treatment combination was replicated three times within each main plot, ensuring robust statistical analysis of the treatment effects across the different wheat cultivars. Two orthogonal plowings prepared the experimental area, land leveling the soil and dividing it into experimental plots. Nitrogen fertilizer used as ammonium nitrate (33.5% N) was applied in two equal doses. Sowing took place on November 25 for both seasons and all standard agricultural practices recommended for growing wheat crop in the region were followed, except for the specific treatments under investigation. The BFC was obtained from the El-Nubaria Sugar Refining Company in El-Beheira, Egypt. The BFC consists of the discharged precipitated solids from the initial sugar beet washing process, primarily a mixture of fertile soil adhered to the rootlets’ network on the sugar beet root surface. This BFC was sun-dried and left for four months during the summer season with regular stirring before utilizing it in the experiment.

**Table 3:** Physical and chemical properties of sugar beet filter cake

Property	
Bulk density (gm^−3^)	0.94
Moisture content (%)	14
pH (1:10)	7.66
EC (1:10)	1.01
Total nitrogen (%)	0.77
Organic carbon (%)	22.26
Organic matter (%)	12.94
Ash (%)	77.74
C/ratio	16.9
Total phosphorus (%)	0.33
Total Potassium (%)	0.51

### Recorded data

The following ten traits were measured in this study using 10 plants from each plot. Chlorophyll content (SPAD) was measured using a SPAD meter (Konica Minolta Optics Inc., Tokyo, Japan) SPAD measurements were taken at three critical growth stages according to the Zadoks scale: tillering (GS 25–29), stem elongation (GS 31–39), and heading (GS 51–59) and the calculated average was used for analysis [38]. Days to maturity (DM) was recorded in days(When 90% of plants in a plot showed loss of green color from the glumes), and flag leaf area (FLA) was measured in square centimeters (cm²). Plant height (PH) was measured in centimeters (cm) from the ground to the top of the spike of 10 randomly selected plants per plot at harvest. The number of spikes per square meter (NSM) was determined by counting fertile spikes in a one-square-meter area. The number of kernels per spike (NKS) was calculated by counting grains from 10 randomly selected spikes and determining the average. Thousand kernel weight (TKW) was measured in grams (g) for each subplot (Dry weight 12% moisture content). Grain yield (GY) was determined after harvesting and threshing, expressed in tons per hectare (t ha⁻¹). Biological yield (BY) was measured by weighing all above-ground plant material from a designated area within each subplot before threshing (3 m² from the central rows of each plot), expressed in tons per hectare (t ha⁻¹). Harvest index (HI) was calculated using the following formula.

$$\:\mathbf{H}\mathbf{I}=\frac{\mathbf{G}\mathbf{r}\mathbf{a}\mathbf{i}\mathbf{n}\:\mathbf{y}\mathbf{i}\mathbf{e}\mathbf{l}\mathbf{d}\:}{\mathbf{B}\mathbf{i}\mathbf{o}\mathbf{l}\mathbf{o}\mathbf{g}\mathbf{i}\mathbf{c}\mathbf{a}\mathbf{l}\:\mathbf{y}\mathbf{i}\mathbf{e}\mathbf{l}\mathbf{d}}\times\:100$$

### Statistical analysis

An analysis of variance (ANOVA) was performed utilizing a split plot design with three replications, assigning seasons to the main plots, cultivars to the sub-plots, and fertilization treatments to the sub-sub plots. Statistical analyses were conducted utilizing RStudio (version 4.2.0, R Core Team, 2023) [39]. The data obtained showed homogeneity between experimental errors of the two seasons. Thus, the results of both seasons will be discussed combined [40]. The ‘agricolae’ package facilitated the execution of ANOVA and Tukey’s honest significant difference (HSD) test at p ≤ 0.05 for mean comparisons upon detection of significant differences. Correlation analysis was conducted utilizing the ‘corrplot’ package to investigate relationships among the measured traits. Path analysis was performed utilizing the ‘lavaan’ package to assess the direct effects of predictor variables on grain yield. Multivariate analyses encompassed radar charts and Principal Component Analysis (PCA).Prior to PCA, data were standardized using z-score transformation (mean-centered and scaled to unit variance) to account for differences in measurement scales across variables. Radar charts were generated utilizing the ‘fmsb’ package to depict the comparative performance of traits among cultivars and fertilization treatments. PCA was conducted utilizing the ‘factoextra’ and ‘FactoMineR’ packages to examine trait relationships and treatment distributions. Biplots were created to illustrate the relationships among variables and observations. Before conducting path analysis, variables were checked for multicollinearity using variance inflation factors (VIF), with values < 5 considered acceptable. The path model was evaluated using goodness-of-fit indices including the Comparative Fit Index (CFI = 0.94), Tucker-Lewis Index (TLI = 0.91), Root Mean Square Error of Approximation (RMSEA = 0.068), and Standardized Root Mean Square Residual (SRMR = 0.045), all indicating adequate model fit.

## Results

### Variance components of wheat performance under integrated fertilization strategies

The analysis of variance revealed significant differences in agronomic, physiological, and yield traits of wheat under different treatments and conditions (Table 4). Seasonal variations significantly influenced number of days to maturity (DM), plant height (PH), flag leaf area (FLA), SPAD, number of spikes per meter square (NSM), and 1000-kernel weight (TKW) at *p* ≤ 0.05, whereas the remaining traits, including number of Kernels per spike (NKS), grain yield (GY), biological yield (BY), and harvest index (HI) showed insignificant differences between the two seasons of study. Cultivars performance demonstrated varying levels of significance across traits, showing highly significant effects (*p* ≤ 0.001) on DM and PH, and TKW (*p* ≤ 0.01) on TKW, while exhibiting significant impacts (*p* ≤ 0.05) on FLA, NSM, GY, HI. Fertilization treatments emerged as the most influential factor, showing highly significant differences (*p* ≤ 0.001) in most measured parameters including DM, PH, FLA, SPAD, NSM, NKS, GY, BY, and HI.

**Table 4:** Analysis of variance (ANOVA) for agronomic and physiological characters, yield and yield components of wheat under different wheat cultivars, N and organic fertilization treatments over two seasons of study

Source	DF	DM	PH	FLA	SPAD	NSM
Block	2	10.65^ns^	4.38^ns^	62.13^ns^	59.21	175.50 ^ns^
Season (S)	1	729***	1029.34*	74.70*	8436.42*	18518.67*
Error (a)	2	0.19	40.34	3.93	94.71	268.17
Cultivar (C)	2	30.27^ns^	2126.09***	398.63*	82.05^ns^	1038.79*
S x C	2	2.02^ns^	204.25**	23.47^ns^	31.72^ns^	4406.46***
Error (b)	8	15.59	22.57	98.50	42.40	167.50
Fertilization (F)	7	61.66***	254.58***	385.53***	168.63***	13948.84***
S x F	7	8.41**	57.67***	89.85**	26.31^ns^	2099.29***
C x F	14	2.33**	12.28^ns^	35.30^ns^	8.57^ns^	461.35^ns^
S x C x F	14	1.22^ns^	12.56^ns^	25.23^ns^	10.13^ns^	405.84^ns^
Error (c)	84	2.23	13.72	27.32	15.62	436.22

Source	DF	TKW	NKS	GY	BY	HI
Block	2	14.8^ns^	6.12^ns^	0.30^ns^	48.74^ns^	109.41^ns^
Season (S)	1	81.45*	100.59^ns^	6.91^ns^	41.01^ns^	22.30^ns^
Error (a)	2	1.57	14.20	2.32	80.97	129.26
Cultivar (C)	2	128.28**	50.50^ns^	3.69*	1.32^ns^	89.49*
S x C	2	46.65*	31.39^ns^	1.73^ns^	1.85^ns^	34.34^ns^
Error (b)	8	10.54	17.05	0.74	6.16	17.10
Fertilization (F)	7	4.18^ns^	84.98***	15.83***	116.83***	86.58***
S x F	7	19.23^ns^	60.69**	2.88***	11.01*	12.69^ns^
C x F	14	14.73^ns^	27.22*	0.78^ns^	3.75^ns^	14.38^ns^
S x C x F	14	12.03^ns^	21.94^ns^	0.52^ns^	5.15^ns^	32.69^ns^
Error (c)	84	12.99	16.57	0.67	5.68	16.28

*ns* Non-significant. *DM* No. days to maturity, *PH* Plant height, *FLA* Flag leaf area, *SPAD* Chlorophyll content, *NSM* Number of Spikes per square meter, *TKW* Thousand kernel weight, *NKS* Kernels per spike, *GY* Grain yield, *BY* Biological yield, *HI* Harvest index

*, **, *** Significant at p≤0.05, p≤0.01, and p≤0.001, respectively

Regarding interaction effects, the season × cultivar interaction showed significant influence on multiple traits: PH (*p* ≤ 0.01), NSM (*p* ≤ 0.001) and TKW (*p* ≤ 0.05). The season × fertilization interaction demonstrated significant effects on all studied traits, except for SPDA, TKW, and HI, showing insignificant differences (p *≥* 0.05). The cultivar × fertilization interaction showed significant differences for DM (*p* ≤ 0.01) and NKS (*p* ≤ 0.05). Notably, the three-way interaction (season × cultivar × fertilization) did not exhibit significant effects on any of the measured traits.

### Effect of growing seasons on agronomic and physiological characters, yield and yield components

Also, as shown above, significant variations between the two growing seasons were demonstrated for several studied traits in bread wheat (Table 5). DM and plant height showed significant differences between seasons, with higher values recorded in the first season (144.54 days and 111.92 cm, respectively) compared to the second season (140.04 days and 106.57 cm, respectively). For FLA, insignificant differences between seasons were detected, as evidenced by the similar medians between the first (39.52 cm²) and second (40.96 cm²) seasons. In contrast, SPAD showed a marked decrease in the second season (25.61) compared to the first season (40.92), with distinct separation between the seasonal distributions. Among yield components, NSM demonstrated significant seasonal variation, with higher values in the first season (319.22) compared to the second season (296.94). For both TKW and NKS, comparable distributions between seasons were recorded, as the second season recorded TKW (57.19 g) and NKS (45.89) higher than those obtained for the first season for TKW (55.69 g) and NKS (44.22). Similarly, GY, BY, and HI displayed insignificant seasonal differences, with substantial overlap in their distributions between the two growing seasons. HI displayed insignificant seasonal differences, recording 36.57 and 37.36% for the first and second seasons, respectively.

**Table 5:** Mean performance of ten agronomic characters, yield and yield components of wheat under different wheat cultivars, N and organic fertilization treatments over two seasons of study

Trait	DM (days)	PH (cm)	FLA (cm)	SPAD	NSM	TKW (g)	NKS	GY(T/ha)	BY(T/ha)	HI %
Season (S)										
First	144.54 a	111.92 a	39.52 a	40.92 a	319.22 a	55.69 b	44.22 b	7.00 b	19.17 b	36.57 a
Second	140.04 b	106.57 b	40.96 a	25.61 b	296.54 b	57.19 a	45.89 a	7.44 a	20.24 a	37.36 a
Cultivar (C)										
sids14	143.21 a	114.15 a	43.43 a	32.36 b	311.60 a	57.10 a	45.14 ab	7.27 ab	19.87 a	36.94 ab
Misr3	141.85 b	101.67 c	39.46 b	34.77 a	302.67 a	54.58 b	43.99 b	6.92 b	19.71 a	35.61 b
Sakha95	141.81 b	111.92 b	37.82 b	32.68 ab	309.38 a	57.64 a	46.03 a	7.47 a	19.54 a	38.34 a
Fertilization (F)										
T1	139.83 d	104.17 b	33.07 c	28.22 b	276.94 de	56.02 a	44.61 ab	6.61 d	16.96 de	38.97 ab
T2	141.33 cd	110.00 a	41.52 ab	34.64 a	305.56 bc	56.06 a	45.96 a	7.12 cd	18.22 cde	39.19 ab
T3	142.78 bc	111.61 a	42.86 ab	35.30 a	324.56 ab	56.93 a	47.28 a	7.84 abc	20.89 abc	37.70 abc
T4	144.33 ab	112.89 a	43.58 ab	36.53 a	340.61 a	56.83 a	47.24 a	8.48 a	21.66 ab	39.38 a
T5	139.89 d	102.78 b	34.25 c	29.09 b	264.22 e	55.79 a	40.86 b	5.56 e	15.79 e	35.59 abc
T6	142.44 bc	109.78 a	37.89 bc	32.79 ab	292.89 cd	56.37 a	43.91 ab	6.62 d	19.79 bcd	33.75 c
T7	142.89 bc	111.00 a	43.07 ab	34.04 a	322.89 ab	56.39 a	44.00 ab	7.52 bc	20.88 abc	36.61 abc
T8	144.33 a	112.22 a	45.66 a	35.53 a	335.39 a	57.13 a	46.56 a	8.00 ab	23.44 a	34.52 bc

Different letters indicate significant differences among treatments (*p* ≤ 0.05) according to Tukey’s HSD

### Performance variation among bread wheat cultivars for agronomic characters, yield and yield components

Analysis of the three bread wheat cultivars revealed significant differences in several agronomic and yield-related traits (Table 5). A minimal variation was showed in DM among cultivars, where Sids 14 recorded slightly higher values (143.21 days) compared to Misr 3 and Sakha 95 (both 141.85 days). Also, PH exhibited significant differences, with Sids 14 attaining the greatest height (114.15 cm), followed by Sakha 95 (111.92 cm), while Misr 3 showed the shortest stature (101.67 cm). In addition, FLA demonstrated significant variability among cultivars, with Sids 14 exhibiting the largest leaf area (43.43 cm²), followed by Misr 3 (39.46 cm²), and Sakha 95 showing the smallest area (37.82 cm²). Leaf chlorophyll content (SPAD) values were the highest in Misr 3 (34.77), while Sakha 95 and Sids 14 were recorded the lowest values by (32.68) and (32.36), respectively. Regarding yield components, NSM was comparable among cultivars, with Sids 14 showing a slight advantage (311.60) over Sakha 95 (309.38), and Misr 3 (302.67). Significant differences were displayed in TKW as Sids 14 and Sakha 95 which produced higher grain weight (57.64 g and 57.10 g, respectively) compared to Misr 3 (54.58 g). In addition, a moderate variation was exhibited in NKS where Sakha 95 exhibited the highest value (46.03), detecting insignificant differences from Sids 14 (45.14). For GY, the highest value was recorded by Sakha 95 (7.47 t/ha), followed by Sids 14 (7.27 t/ha), while Misr 3 produced the lowest yield (6.92 t/ha). A minimal variation was exhibited in BY among the three wheat cultivars, ranging from 19.54 t/ha (Sids 14) to 19.87 t/ha (Misr 3). Also, HI revealed significant differences, where Sakha 95 showed the highest value (38.34%), followed by Sids 14 (36.94%), while Misr 3 recorded the lowest value of HI (35.61%).

### Interaction effects of nitrogen rates and sugar beet filter cake

The application of different combinations of organic BFC and N fertilization rates significantly affected the studied traits (Table 5). For DM, significant variation among the combined treatments of organic and N fertilization was revealed, with the highest value (144.33 days) was recorded under both T4 and T8 treatments, while the lowest values were observed under T1 and T5 treatments (139.83 and 139.89 days, respectively). There was a positive response between PH and increasing N rates, the highest values were recorded under T4 (112.89 cm) and T8 (112.22 cm) treatments. The lowest PH was observed under T5 (102.78 cm) and T1 (104.17 cm) treatments, indicating the significant role of N in vegetative growth. Furthermore, FLA showed marked differences among combined fertilization treatments, the maximum leaf area was achieved under T8 (45.66 cm²), followed by T4 (43.58 cm²). The minimum FLA was recorded under T1 (33.07 cm²). For leaf chlorophyl content, the treatments receiving higher N rates and combined with organic fertilization (T4) showed significantly higher values of (36.53), while the treatments T1 and T5 recorded the lowest values of (28.22 and 29.09, respectively). A positive response was noticed between NSM and increased N rates, as the treatments T4 and T8 treatments which produced the highest values (340.61 and 335.39 spikes/m², respectively). The lowest NSM was recorded under T5 (264.22 spikes/m²). Also, TKW showed minimal variation among treatments, ranging from 55.79 g (T5) to 57.13 g (T4). For NKS the treatments T3 and T4 were recorded at the highest levels of 47.28 and 47.24, respectively, while the lowest value was observed under T5 treatment (40.86). A noticeable response to fertilization treatments was shown for GY, with T4 producing the highest yield (8.48 t/ha), followed by T8 (8.00 t/ha), while T5 resulted in the lowest yield (5.56 t/ha). A similar trend to GY was observed in BY, with maximum yield under T8 (23.44 t/ha) and T4 (21.66 t/ha) treatments, and minimum under T5 treatment (15.79 t/ha). HI showed some variation among treatments, T4 recording the highest value (39.38%), while T6 showed the lowest value of (33.75%).

### Trait associations and path analysis of yield components in bread wheat

**Fig. 1:**
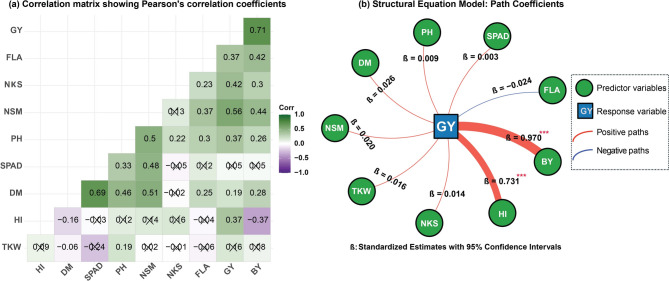
Correlation coefficients and path analysis of ten studied traits (agronomic and physiological characters, yield and yield components) in bread wheat under organic and inorganic fertilization treatments across two growing seasons. **a** Correlation matrix shows Pearson’s coefficients (r) among ten traits. Color intensity indicates correlation strength and direction (green = positive, purple = negative). Values without ‘X’ are insignificant (P *≥* 0.05). **b** Path coefficient diagram showing direct effects of nine predictor variables on grain yield (GY). Arrow thickness indicates effect strength (red = positive, blue = negative). Asterisks denote significance levels: *P ≤ 0.05, **P ≤ 0.01, ***P ≤ 0.001. Analyzed traits include No. of days to maturity (DM), plant height (PH), flag leaf area (FLA), SPAD, spikes per square meter (NSM), thousand kernel weight (TKW), kernels per spike (NKS), grain yield (GY), biological yield (BY), and harvest index (HI)

The correlation analysis and path coefficient study revealed complex interrelationships among the studied traits and their influence on GY in bread wheat (Fig. 1a and b). For the correlation analysis (Fig. 1a), strong positive correlations were observed between GY and several traits. GY showed the strongest positive correlation with BY (*r* = 0.71), followed by NSM (*r* = 0.56), and NKS (*r* = 0.42). Also, SPAD and DM exhibited high positive correlation (*r* = 0.69). Several significant positive correlations were exhibited between NSM and each of DM (*r* = 0.51), PH (*r* = 0.50), and SPAD (*r* = 0.48). Also, BY showed a positive and significant correlation with NSM (*r* = 0.44). Notably, negative and significant correlations were also detected between each of BY and HI (*r* = −0.37) and DM and HI (*r* = −0.37). The path analysis (Fig. 1b) revealed the direct effects of various traits on the response variable GY. The results showed that BY exhibited the strongest direct positive effect on GY (0.970***), underscoring its critical role in determining final yield. HI showed the second strongest direct effect (0.731***), highlighting its significant effect on yield determination despite its relatively weak correlation with GY. All the remaining predictors showed insignificant and low effects on the final yield (GY), including those with positive effect such as DM and NSM with coefficients of 0.026 and 0.020. The only negative effect was displayed by FLA with coefficient of −0.024.

### Multivariate assessment of the studied traits response to different cultivars and fertilization treatments

**Fig. 2:**
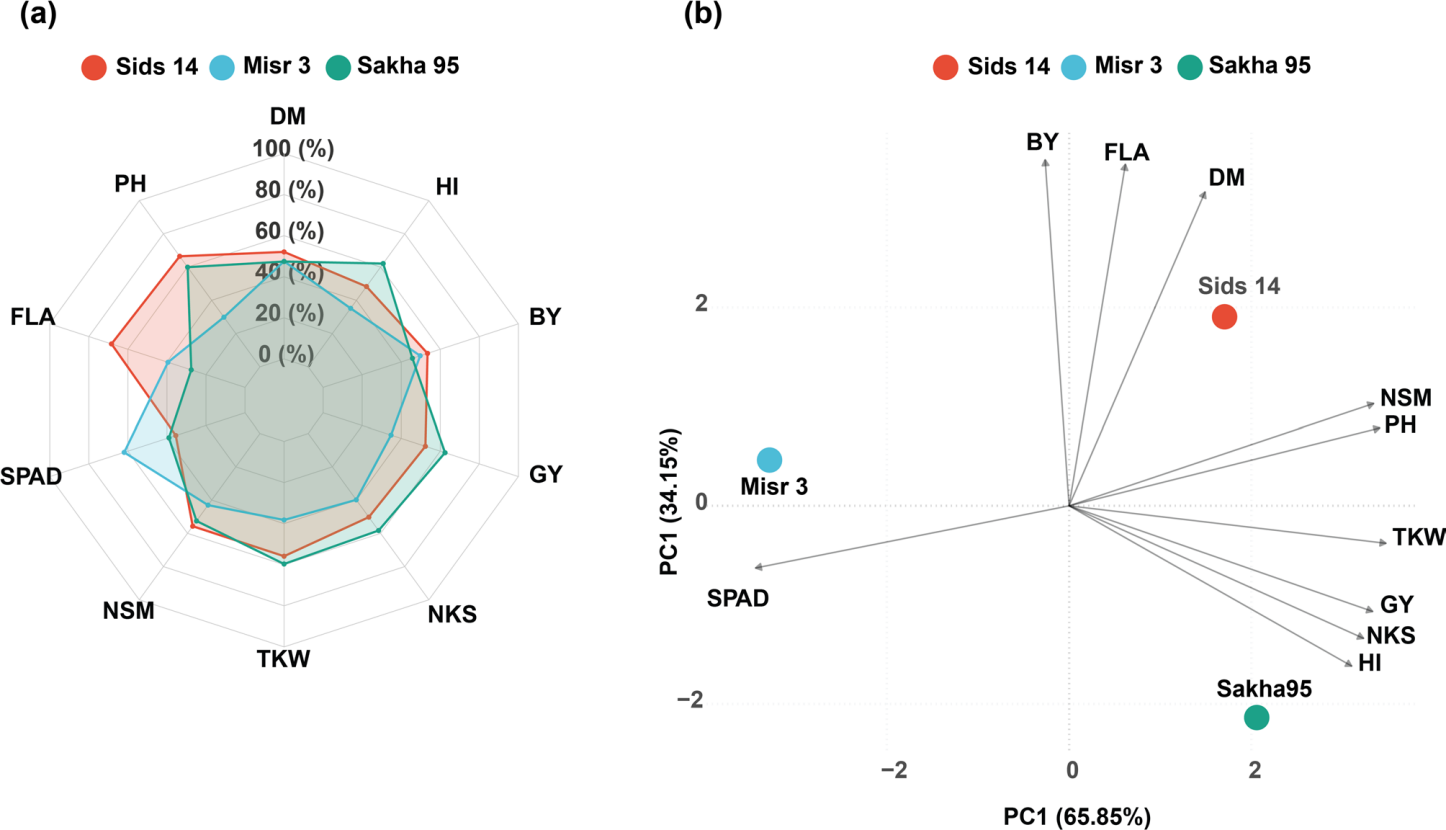
Multivariate characterization of the studied traits in three wheat cultivars. **a** A radar chart illustrates the comparative performance of ten agronomic traits (DM, PH, FLA, SPAD, NSM, TKW, NKS, GY, BY, and HI) across three wheat cultivars (Sids 14, Misr 3, and Sakha 95). **b** PCA biplot shows the distribution of cultivars and trait relationships. The first two principal components explained 100% of total variation (PC1 = 65.85%, PC2 = 34.15%). Vectors represent trait loadings and their relationships with cultivar performance

Based on three wheat cultivars, the multivariate analysis revealed distinct patterns of trait expression and genotypic variation (Fig. 2a and b). The radar chart analysis demonstrated unique trait profiles for Sids 14, Misr 3, and Sakha 95, reflecting their differential performance across measured parameters. The analysis showed that Sakha 95 showed superiority for TKW, NKS, GY and HI, whereas Sids 14 indicated high performance in respect to PH, FLA, DM, and BY, while Misr 3 indicated high performance in respect SPAD values. The polygons formed by trait values showed overlapping yet distinguishable patterns among cultivars, indicating genotype-specific trait expressions.

For the results of PCA analysis, the results showed that there was a clear differentiation among the three cultivars based on their agronomic performance, with the first two principal components explaining 100% of the total variation (PC1 = 65.85%, PC2 = 34.15%). The PCA biplot revealed a clear spatial separation of the three cultivars, indicating their distinct agronomic characteristics. The results of biplot also pinpointed that Sids 14 was positioned in the upper right quadrant, showing strong associations with BY, FLA, NSM, and DM. Also, Sakha 95 was located in the lower right quadrant, demonstrating positive associations with TKW, GY, NKS, and HI. In contrast, Misr 3 was positioned in the left quadrant, showing relatively lower values for most traits, except for its high records in SPAD. In addition, the vector analysis from the PCA biplot revealed important trait relationships and their contributions to cultivar differentiation. A cluster of traits including NSM, PH, TKW, GY, NKS, and HI showed similar directional patterns, suggesting their coordinated expression. BY, DM, and FLA vectors were closely aligned but in a different direction, indicating their distinct contribution to cultivar performance. The SPAD vector showed a unique orientation, suggesting its independent contribution to cultivar variation.

**Fig. 3:**
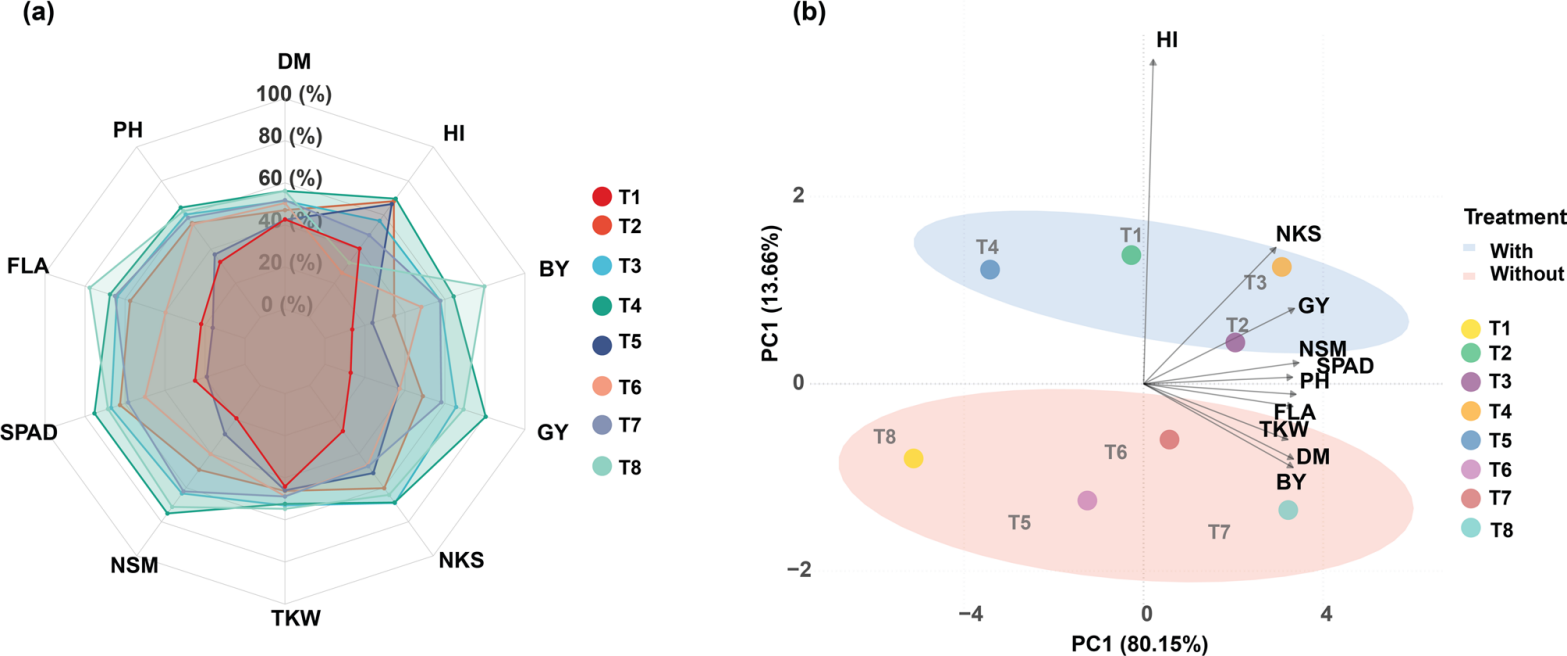
Multivariate analysis of wheat studied traits under different fertilization treatments. **a** Radar chart showing the relative performance of ten agronomic traits (DM, PH, FLA, SPAD, NSM, TKW, NKS, GY, BY, and HI) across eight fertilization treatments. The polygons represent treatment performance with different fertilizer rates (60, 120, 180, and 240 kg ha⁻¹) under with and without fertilization conditions. **b** PCA biplot displaying the relationships among traits and treatment distributions. The first two principal components explained 93.81% of total variation (PC1 = 80.15%, PC2 = 13.66%). Blue and pink ellipses indicate the clustering of with and without fertilization treatments, respectively. Vectors represent trait loadings and their relationships

Based on different fertilization combinations of treatments of N and organic, the multivariate analysis of agronomic traits revealed distinct patterns of trait expression and treatment effects (Fig. 2a-b). The radar chart analysis demonstrated that fertilizer applications substantially influenced the expression of key agronomic traits in wheat (Fig. 3a). Treatments which combine N fertilizers with BFC as organic matter, including those of highest N does of 240 kg ha⁻¹, exhibited consistently superior performance across all measured parameters compared to their counterparts with no organic amendments applied. Notably, the higher N fertilization rates (240 kg ha⁻¹ and 180 kg ha⁻¹) generated the most expansive polygons in the radar plot, indicating enhanced overall agronomic performance. In contrast, the lowest N treatment with non-organic fertilizer applied (T1) showed the lowest record in respect to all of the parameters studied.

The findings of PCA provided further insights into the relationships among traits and treatment responses. The first two principal components collectively explained 93.81% of the total variation, with PC1 accounting for 80.15% and PC2 explaining 13.66%. Similar to cultivars-based PCA, the PCA biplot revealed a clear discrimination between combined N-organic fertilization and those N treatments without organic applications along PC1, with those with organic fertilizations treatments clustering in the upper right quadrant and those having non-organic fertilization treatments grouping in the lower left quadrant. The vector analysis from the PCA biplot revealed significant associations among agronomic traits. Based on that differentiation due to the different 8 combination treatment of N-organic fertilization treatments, GY showed strong positive associations with NSM and SPAD, suggesting these traits as key determinants of performance under varying fertilization regimes. HI and NKS exhibited close directional alignment, indicating their synchronized response to fertilization treatments. DM, BY, TKW and FLA displayed similar directional influences. Overall, these treatment-specific responses indicated that higher fertilization rates, particularly 240 kg ha⁻¹ and 180 kg ha⁻¹, were positioned proximally to yield-related trait vectors, demonstrating their positive influence on these important agronomic parameters. In contrast, the lowest N fertilization rate (60 kg ha⁻¹) and non-organic fertilization treatments were positioned opposite to most trait vectors, indicating suboptimal performance across most of measured traits.

## Discussion

### Effects of integrated nutrient management on wheat performance

Our study demonstrated significant benefits of integrating sugar beet filter cake with mineral nitrogen fertilization on wheat productivity in newly reclaimed calcareous soils. The analysis of variance revealed that fertilization treatments had the most substantial influence on crop performance, with highly significant effects (*p* ≤ 0.001) on 90% of the assessed parameters (Table 3). This strong treatment response highlights the critical role of nutrient management in determining wheat performance in nutrient-limited calcareous soils, which typically have low organic matter content (0.24–0.31%, Table 1) and high calcium carbonate levels (22.92–23.82%). The superior performance of treatments combining sugar beet filter cake with mineral nitrogen was evident in multiple yield parameters. The T4 treatment (240 kg N ha⁻¹ with filter cake) produced the highest grain yield (8.48 t ha⁻¹), representing a 6% yield advantage over the equivalent nitrogen rate without organic amendment (T8: 8.00 t ha⁻¹). This yield enhancement aligns with meta-analyses by Zhang et al. [41] and Kebalo et al. [42], who reported average yield increases of 5–10% when organic amendments complemented mineral fertilization in similar soil types. The positive response to integrated nutrient management was consistent across multiple yield components. Treatments with filter cake application showed higher spikes per square meter (340.61 in T4 vs. 335.39 in T8) and kernels per spike (47.24 in T4 vs. 46.56 in T8), suggesting improvements in both tillering capacity and reproductive efficiency. Similar enhancement patterns were observed in physiological parameters, with treatments receiving filter cake maintaining higher SPAD values throughout the growing period. This aligns with findings by Mohanty et al. [43], who reported sustained nitrogen availability and improved photosynthetic efficiency when organic amendments complemented mineral fertilizers. The magnitude of yield response to integrated nutrient management varied with nitrogen rates, following a pattern consistent with resource optimization theory [44]. At the lowest nitrogen rate (60 kg N ha⁻¹), the relative yield increase from filter cake addition was substantial (18.9%, comparing T1 vs. T5), indicating that organic inputs partially compensated for mineral nitrogen deficiency. At intermediate rates (120–180 kg N ha⁻¹), yield increases remained significant (7.6–8.2%), while at the highest rate (240 kg N ha⁻¹), the relative advantage diminished to 6.0%. This pattern suggests that as mineral nitrogen approaches sufficiency, the additional benefits of organic amendments become more associated with non-nitrogen factors such as improved soil physical properties or enhanced micronutrient availability rather than nitrogen substitution.

Our findings indicate that integrated nutrient management allows for potential reduction in mineral nitrogen requirements. The T3 treatment (180 kg N ha⁻¹ with filter cake) produced statistically equivalent yields (7.84 t ha⁻¹) to the T8 treatment (240 kg N ha⁻¹ without filter cake, 8.00 t ha⁻¹), suggesting that filter cake application could potentially reduce mineral nitrogen inputs by approximately 60 kg N ha⁻¹ (25%) without compromising productivity. This nitrogen input reduction represents a significant economic and environmental benefit, considering that nitrogen fertilizer typically constitutes 30–40% of production costs in wheat cultivation in the region [45, 46].

### Cultivar-specific responses to fertilization strategies

The three wheat cultivars in our study exhibited distinct responses to integrated nutrient management, highlighting the importance of genotype-specific recommendations for fertilization practices. Sakha 95 consistently demonstrated superior grain yield (7.47 t ha⁻¹) and harvest index (38.34%) across treatments, suggesting more efficient partitioning of assimilates to grain production compared to the other cultivars. This cultivar also showed the strongest positive response to filter cake application at high nitrogen rates, with a 7.5% yield increase under the T4 treatment compared to T8. Sids 14 exhibited optimal morphological characteristics, including maximum plant height (114.15 cm) and flag leaf area (43.43 cm²), along with good yield potential (7.27 t ha⁻¹) but relatively moderate response to filter cake application (5.2% yield increase under T4 vs. T8). These results suggest that cultivars with more extensive vegetative development may benefit less from organic amendments in terms of relative yield enhancement, possibly due to their inherently greater resource capture capacity. Misr 3, despite showing the highest chlorophyll content (SPAD values of 34.77), produced the lowest grain yield (6.92 t ha⁻¹) and harvest index (35.61%), indicating less efficient conversion of photosynthates to grain. This cultivar showed the least consistent response to filter cake application across nitrogen rates, suggesting potential genotype-specific limitations in utilizing additional resources provided by organic amendments.

The cultivar × treatment interaction, while not statistically significant for grain yield (Table 4), showed notable trends in the relative ranking of cultivars across treatments. Sakha 95 maintained yield superiority under both integrated and conventional fertilization regimes, but its advantage over other cultivars was more pronounced under integrated nutrient management. These patterns align with findings by El-Sorady et al. [47], who reported cultivar-specific variation in response to diverse nutrient sources based on genetic differences in nutrient use efficiency and root architecture.

The multivariate analysis further elucidated cultivar differentiation patterns. The PCA biplot (Fig. 2b) showed clear spatial separation of the three cultivars, with the first two principal components explaining 100% of the total variation (PC1 = 65.85%, PC2 = 34.15%). Sakha 95 was strongly associated with yield-related traits (TKW, GY, NKS, and HI), while Sids 14 showed stronger associations with vegetative growth parameters (BY, FLA, NSM, and DM). This distinct multivariate profiling suggests that breeding programs should consider selection under diverse nutrient supply mechanisms rather than conventional high-input conditions alone, as genetic potential may be expressed differently under integrated management systems.

### Mechanisms underlying synergistic effects of Organic-Mineral integration

The positive effects of integrating BFC with mineral nitrogen observed in our study can be attributed to several complementary mechanisms. The gradual nutrient release pattern from organic sources likely improved synchronization between nutrient availability and crop demand. The BFC used in our study had a C: N ratio of 16.9 (Table 2), which is within the optimal range for net nitrogen mineralization without causing significant immobilization [4, 48]. Based on mineralization kinetics of similar materials reported by Roig et al. [26], approximately 20–25% of total organic nitrogen would become plant-available within 30 days after incorporation, with an additional 15–20% mineralized during the subsequent 60–90 days. This release pattern aligns favorably with wheat’s nutrient demand curve, which shows moderate N requirements during early vegetative stages and peak demand during stem elongation and heading [49, 50]. BFC application likely enhanced nutrient retention and reduced losses compared to sole mineral fertilization. The higher organic matter content contributed by filter cake (12.94%, Table 3) improves soil cation exchange capacity, which is particularly valuable in sandy calcareous soils with inherently low nutrient retention capacity. Studies in similar environments have shown that organic amendments can reduce nitrate leaching by 30–40% [51, 52] and ammonia volatilization by 20–25% [53] compared to mineral fertilization alone, potentially explaining the higher nitrogen use efficiency observed in our integrated treatments. The addition of BFC may have improved soil physical properties, enhancing root development and nutrient accessibility. Although not directly measured in our study, previous research with similar materials has documented 15–20% increases in water holding capacity and 25–30% reductions in bulk density following application of sugar industry by-products to calcareous soils [35, 54]. Such improvements in soil physical properties are particularly beneficial in the sandy clay loam soils of our experimental site, potentially facilitating more extensive root exploration and greater access to both water and nutrients. The BFC may have contributed additional nutrients beyond nitrogen. With 0.33% phosphorus and 0.51% potassium (Table 2), the application of 24 t ha⁻¹ filter cake contributed approximately 79 kg P and 122 kg K per hectare. In calcareous soils, organic-bound phosphorus often shows enhanced availability compared to mineral sources, as the organic acids produced during decomposition can temporarily reduce soil pH in microenvironments and increase phosphorus solubility [55–58].

Organic carbon input may have stimulated soil biological activity, enhancing nutrient cycling and possibly producing plant growth-promoting substances [59–61]. Maffia et al. [62] demonstrated that organic amendments can enhance root growth, increase nutrient absorption, and improve photosynthetic efficiency through both direct nutrient contributions and indirect effects on soil biological communities and plant physiology. The path analysis provided further insights into yield determination, revealing that biological yield (direct effect = 0.970***) and harvest index (direct effect = 0.731***) were the primary determinants of grain yield (Fig. 1b). This suggests that integrated nutrient management enhanced yield primarily by increasing overall biomass production while maintaining efficient partitioning to grain balanced effect that maximizes productivity. The high positive correlation between biological yield and number of spikes per square meter (*r* = 0.44) indicates that enhanced tillering capacity was a key mechanism by which filter cake application improved overall productivity.

### Practical implications for sustainable wheat production

Our findings have several important implications for sustainable wheat production in newly reclaimed calcareous soils. First, the integration of BFC at 24 t ha⁻¹ with mineral nitrogen fertilization offers a viable strategy for optimizing wheat productivity while potentially reducing environmental impacts. The equivalent yield performance between T3 (180 kg N ha⁻¹ with filter cake) and T8 (240 kg N ha⁻¹ without BFC) suggests that farmers could reduce mineral nitrogen inputs by approximately 25% through integrated nutrient management without compromising yield. Second, the timing of filter cake application appears critical for maximizing benefits. In our study, BFC was incorporated two weeks before sowing, allowing initial microbial processing before peak crop nutrient demand. This timing appears suitable for wheat production in the region, although future research could explore split applications of organic amendments to further enhance synchronization with crop demand throughout the growing cycle. Third, cultivar selection should be considered alongside fertilization strategy. Based on our results, Sakha 95 would be the preferred cultivar when BFC is available due to its superior response to integrated nutrient management, particularly at higher nitrogen rates. For systems relying solely on mineral fertilization, the choice between Sakha 95 and Sids 14 may depend more on other agronomic considerations such as disease resistance or drought tolerance. Fourth, the regional availability of sugar beet filter cake as an industrial by-product represents an opportunity for creating circular nutrient flows within agricultural systems. With sugar beet production in Egypt rising from 7.84 to 14.83 million metric tons between 2010 and 2021 (Ministry of Agriculture, 2022), the volume of available filter cake is substantial. Directing this material back to agricultural fields not only provides agronomic benefits but also addresses waste management challenges for the sugar industry.

Finally, while our study focused on immediate agronomic benefits, integrated nutrient management likely provides longer-term soil health improvements. The gradual build-up of soil organic matter through repeated application of filter cake could progressively enhance soil quality, potentially leading to cumulative yield benefits over multiple cropping cycles [63, 64]. Long-term studies in similar environments have documented sustained improvements in soil structure, water retention, and biological activity following consistent application of organic amendments [18, 65–67]. It is important to note, however, that the optimal application rate of filter cake may vary depending on soil conditions, cultivar choice, and environmental factors. The 24 t ha⁻¹ rate used in our study represents a balance between agronomic benefits and practical application considerations, but site-specific adjustments may be necessary based on soil testing and economic constraints. Future research should explore varying application rates, particularly under different soil texture conditions that are common in newly reclaimed areas of Egypt.

## Conclusion

This research provides evidence for the potential benefits of integrating BFC with mineral nitrogen fertilization in wheat production systems on newly reclaimed calcareous soils. The analysis of agronomic performance across two growing seasons revealed significant effects of fertilization treatments on most measured parameters, with treatments combining BFC and higher nitrogen rates (180–240 kg N ha⁻¹) generally showing improved growth and yield metrics. Among the cultivars studied, Sakha 95 exhibited superior grain yield (7.47 t ha⁻¹) and harvest index (38.34%), while Sids 14 demonstrated better morphological characteristics including plant height and flag leaf area. The path coefficient analysis identified biological yield and harvest index as the primary determinants of grain yield, with direct effect coefficients of 0.813*** and 0.614***, respectively. Principal component analysis differentiated treatments with and without organic fertilization, accounting for 93.81% of total variation, suggesting distinct response patterns to different nutrient management approaches. The positive response to combined application of sugar beet residues with mineral nitrogen indicates that integrated nutrient management may offer a viable strategy for optimizing resource use in wheat cultivation on calcareous soils. However, the limited significant interactions between cultivars and fertilization treatments for most traits suggest that cultivar selection and fertilization strategies could be optimized independently.

## Data Availability

“The current study did not involve the generation of new sequencing data. The datasets generated and analyzed during the current study are available from the corresponding author upon reasonable request.”
